# Spontaneous coronary artery dissection and vascular Ehlers-Danlos syndrome: a systematic review and case series

**DOI:** 10.1038/s41431-026-02074-1

**Published:** 2026-03-17

**Authors:** Neeti Ghali, Chloe Angwin, Samuel Liebert, Andrew Deaner, Katherine von Klemperer, Nigel Wheeldon, Diana Johnson, Glenda Sobey, Nilesh J. Samani, Tom R. Webb, Ania Baranowska, Anju Velvet, Sylvia Keigwin, Duncan Baker, Katherine Read, Fleur Stephanie van Dijk, David Adlam

**Affiliations:** 1 National EDS Service London, London North West University Healthcare NHS Trust, Harrow, London, UK; 2 Department of Metabolism, Digestion and Reproduction. Section of Genetics and Genomics. Imperial College London, London, UK; 3 Department of Cardiology, Barking, Havering and Redbridge University NHS Trust, London, UK; 4 Department of Cardiology, Barts Heart Centre, Barts Health NHS Trust, London, UK; 5 Cardiothoracic Centre, Northern General Hospital, Sheffield Teaching Hospitals NHS Trust, Sheffield, UK; 6 EDS National Diagnostic Service, Sheffield Children’s NHS Foundation Trust, Sheffield, UK; 7 Leicester British Heart Foundation Centre of Research Excellence, Department of Cardiovascular Sciences and NIHR Biomedical Research Centre, University of Leicester, Leicester, Leicestershire UK; 8 Sheffield Diagnostic Genetics Service, Sheffield Children’s NHS Foundation Trust, Sheffield, UK; 9 Department of Psychiatry, West London NHS Trust, London, UK

**Keywords:** Myocardial infarction, Clinical genetics

## Abstract

Spontaneous coronary artery dissection (SCAD) is a cause of acute myocardial infarction predominantly affecting adult women. A proportion of SCAD cases are associated with rare heritable connective tissue disorders. Vascular EDS (vEDS), due to deleterious variants in *COL3A1*, is one of the most common of these. Our aim was to identify specific features of SCAD in vEDS which may aid patient selection for genetic testing. A systematic review of published cases of individuals with SCAD and vEDS was conducted. Additionally, patients with SCAD and genetically confirmed vEDS (SCAD-vEDS) were identified through the UK national EDS service and UK SCAD registry. Data were collected on presentation, management and extra-cardiac findings. Angiography was compared with an age and sex-matched, exome sequenced, control cohort with SCAD but without vEDS (SCAD-nonvEDS). Data from ten SCAD-vEDS patients were identified. There was a lower average age of SCAD and higher proportion of males in individuals with SCAD-vEDS, however differences should be interpreted carefully given cohort size. Fifty-six cases of SCAD-vEDS were identified through systematic review. Systemic features were present in most but not all cases. This report presents a new, angiographically characterised case-control cohort along with a systematic review of the current literature. Whilst clinical differences appear between the SCAD-vEDS and SCAD-nonvEDS groups, these are insufficient to accurately distinguish SCAD-vEDS from the general SCAD population. All individuals with SCAD should be evaluated for underlying vEDS but clinical assessment will miss some cases. Wider genetic testing in some SCAD patients may be merited to enable appropriate management. Systematic review registration: https://www.crd.york.ac.uk/prospero/536751 Identifier: 536751.

## Introduction

Spontaneous coronary artery dissection (SCAD) is a rare cause of acute myocardial infarction (AMI). Previously, SCAD was considered a multifactorial disorder affecting young women with no clear risk factors and mainly occurring in the peripartum period or as an unusual complication of a heritable connective tissue disorder [1]. More recently, large observational studies have demonstrated the population affected by SCAD is broader [2]. SCAD is primarily diagnosed by coronary angiography supported by intra-coronary imaging. Any coronary artery may be affected, but the left anterior descending (LAD) is most frequently involved. While many SCAD cases are likely to be the result of polygenic risk [3], gene sequencing studies in SCAD have identified a small subset of cases that are associated with single-gene disorders, primarily hereditary connective tissue disorders [4, 5]. One of the commonest associated hereditary connective tissue disorders is vascular Ehlers-Danlos Syndrome (vEDS).

vEDS is one of 13 described types of Ehlers-Danlos Syndrome (EDS), a group of heritable connective tissue disorders [6]. vEDS is characterized by predisposition to arterial and hollow organ rupture resulting in life-threatening events [7, 8], It is an autosomal dominant disorder caused primarily by deleterious heterozygous variants in the *COL3A1* gene which encodes the alpha-1 chain of type III procollagen [9]. Most deleterious variants in *COL3A1* result in tissue fragility by affecting the incorporation of the (pro)collagen alpha-1 chains into a triple helical structure, leading to up to 87.5% abnormality in type III (pro)collagen which is expressed in the vessels walls and hollow organs [10]. Any diagnostic or therapeutic endovascular or surgical intervention in individuals with vEDS is considered extremely carefully given the significant high risk of complications due to tissue fragility [11].

Although earlier studies describe a poorer outcome with significantly decreased life expectancy for individuals with vEDS [12], more recent long-term observational studies describe a lower annual occurrence of arterial complications, longer lifespan and a higher survival rate, especially in the presence of regular medical care [13–15]. A 2019 study looking specifically at arterial events in individuals with vEDS identified the mesenteric arteries, followed by cerebrovascular, iliac and renal arteries to be the most affected arteries, and found that the majority of complications were managed medically rather than with surgical intervention [16].

At present, it is not known if patients with vEDS within the general SCAD population can be accurately identified on the basis of their clinical phenotype. A recent research letter commented on the variable phenotype in vEDS patients, and the difficulty but importance of distinguishing this group from other individuals with SCAD. vEDS related generalised tissue fragility warrants tailored, up-to-date management which can likely improve life expectancy significantly and avoid potential risks related to procedures [17]. Therefore, being able to distinguish this patient group could have significant benefits both for affected individuals and medical health care professionals involved in their care. Our objective in this study was to assess for potential clinical and angiographic findings in SCAD patients that would suggest a diagnosis of vEDS. For this purpose, (i) data from a systematic review of the literature regarding SCAD occurrence in vEDS was analysed as well as (ii) data from a cohort of patients with SCAD and a molecularly confirmed diagnosis of vEDS (SCAD-vEDS) compared to age and sex-matched controls with SCAD but without a vEDS diagnosis (SCAD-nonvEDS).

## Methods

### Systematic review

A systematic search of the literature was conducted to identify published data on SCAD and vEDS to establish all possible reported cases of SCAD and vEDS occurring together. Further details including PRISMA figure are summarised in supplementary information (S1). Database search was performed from 1994 (molecular cause of vEDS known) until Sept 2024. Data extraction and analysis was performed by two investigators (SL and CA) independently, using the systematic review software Covidence [18], and search strings were used to examine electronic databases for relevant references (available in supplementary materials (S1.1)). Duplicates were removed and search results were initially screened by title and abstract and those without clear associations with SCAD and vEDS were removed. Reference lists of included studies were screened for additional studies. After identifying potential articles, the two investigators convened. Articles or single cases were excluded if: (i) they did not contain sufficient information, (e.g. no mention of a (likely) pathogenic *COL3A1* variant, no indication that the SCAD was angiographically confirmed), or (ii) if the cases included individuals in the currently reported cohort, or (iii) where there was a high likelihood that the included individuals had been reported in multiple papers (e.g. papers by the same groups) to avoid replication of data (see supplement 1 for detailed breakdown of excluded papers and cases). Clinical data was extracted from manuscript and supplementary information, and authors were contacted for specific additional information on cases with missing information, but unfortunately no replies were received. The study followed the PROSPERO guidelines and Preferred Reporting Items for Systematic reviews and Meta-Analyses guidelines [19].

### Retrospective cohort study population

Patients were recruited from the UK National EDS Service in London and Sheffield and the UK SCAD Registry. The UK SCAD study was approved by the UK National Research Ethics Service (14/EM/0056) and the UK Health Research Authority and conducted in accordance with the Declaration of Helsinki. All SCAD-vEDS and SCAD-nonvEDS patients gave fully informed written consent to participate in the study.

### Confirmation of SCAD and vEDS diagnosis

Data were analysed for a reported history of SCAD or MI and likely vEDS in patients seen in the EDS services. SCAD registry cases were identified through a group of individuals that underwent genomic sequencing [5]. Demographic information, the patient’s medical history and examination findings and a detailed history of the SCAD event were obtained from the patient’s medical record if it was available. All patients had SCAD confirmed by invasive angiography reviewed by a minimum of two Cardiologists with expertise in SCAD. Patients with coronary artery spasm, atherosclerotic, traumatic or iatrogenic dissection were excluded as were those where only the angiogram report was available. Genetic confirmation of a deleterious *COL3A1* variant was obtained using ACGS criteria [20]. The reference transcript for all *COL3A1* variants was NM_000090.3.

### Angiographic analysis

Angiograms from the SCAD-vEDS cohort were held centrally and these were blindly reviewed by three experienced interventional cardiologists with expertise in SCAD with any initial disagreements resolved by consensus. The control group (SCAD-nonvEDS) consisted of age-matched (to exact year) and sex-matched patients who had had SCAD and in whom no genetic diagnosis had been made from our exome sequencing study [5]. A randomisation algorithm was used to select the controls from the overall exome sequenced cohort at a ratio of two SCAD-nonvEDS to one SCAD-vEDS. Statistical analysis of the angiogram findings in the case control cohort was carried out using a two-tailed Fisher’s exact test. UK SCAD patients are central to all UK SCAD research and the findings of this study will be disseminated via our patient organisation (Beat SCAD—Supporting SCAD Patients and Funding Research). Several individuals reported in this paper are also part of the natural exploration of EDS study (NEEDS) and dissemination of research results will be through the national EDS service. In addition, the UK vascular EDS charity, Annabelle’s Challenge is involved in informing patients about the research and disseminating findings.

## Results

### Systematic review

#### Screening

Initial searches identified 1726 papers, 1050 of which were excluded as duplicates and 7 further studies were added after citation searches. 683 studies underwent initial title and abstract screening, with 583 excluded as not being relevant to this project (i.e. not including vascular EDS and/or not including SCAD). Full text screening was carried out for the remaining 100 papers, and 70 were excluded (see S1.3 for exclusions). Full-text analysis of systematic review of the remaining 30 papers yielded a total of 56 reported cases of confirmed SCAD in individuals with molecularly confirmed vEDS.

#### Data extraction

Of the 30 included papers, six studies concerned cohorts of individuals with SCAD screened for deleterious *COL3A1* variants (11 patients) [5, 21–25]; nine publications (31 patients) were identified from vEDS cohort studies [14–16, 26–31] and thirteen were case reports [22, 32–43]. The largest series [26] looked at a vEDS cohort with 737 vascular events. Out of these, 25 events were related to the coronary artery (21 concluded to be SCAD with one excluded as duplicate).

One publication examined individuals with heritable thoracic aortic disease [31]. (Table 1 and Supplementary Table S1.4). One paper had analysed data from three registries from 24 centres enroling 7568 individuals with thoracic aortic aneurysm; three patients with SCAD and deleterious *COL3A1* variants were identified [44]; however, there is most likely overlap with at least two of these individuals and those from case reports described in Table 1 and Supplementary Table S1.4 and therefore these cases were not included. Of note, where reported, affected arteries included the left main coronary artery (LMCA) *n* = 15, left anterior descending (LAD) (*n* = 15), left circumflex artery (LCx) (*n* = 7) and right coronary artery (RCA) *n* = 15.

**Table 1:** Summary data from systematic review of the literature on SCAD and vascular EDS.

Reference	Number of individuals	Genetic cause	Age/Sex	Family History	Previous history	Presentation/Risk factors	Management/Complications
Nakamura et al. 2008 [34]	1	c.1988G>A (p.Gly663Asp)	33/F	Nil	Yes	4 months post-partum; Bilateral lower jaw pain; angiography showed dissection of posterolateral branch of LCx After 2 weeks – further chest pain; angiography showed LAD dissection and occlusion and RCA dissection	Angioplasty to stent LAD. NB: few hours later, patient died from rupture of right common iliac artery (possibly related to puncture site)
Hampole et al. 2011 [32]	1	c.31+1 G > C	29/M	n/r	Yes	Chest pain Angiography showed aneurysm and dissection of LAD	Saphenous vein bypass graft; Post-op VF, resuscitated including chest compressions. Opening of chest identified an aortic rupture (possibly due to chest compressions). Repaired surgically.
Liestritz et al. 2011 [30]	1/82	*COL3A1* variant not specified	n/r	n/r	n/r	SCAD, no further details	n/r
Ohyama et al. 2011 [26]	1	p(Gly85Asp)?	45/F	n/r	Yes	Sudden onset of chest pain following transarterial embolization for right carotid-cavernous fistula	Extravasation of contrast material from multiple sites in distal segments of the left circumflex artery and right coronary artery, resulting in pericardial bleed and tamponade.
Pepin et al. 2014 [26]	20*: cohort of 1231 vEDS patients	n/r	12x F 8x M	n/r	Variable in cohort	9x CA; 2x LAD; 1x LAD, LM; 4x RCA; 1x RCA, LAD; 1x LCA; RCA; 1x LM; 1x LC; 3 pregnancy-related	7 died
Murray et al. 2014 [27]	1/526	*COL3A1* variant not specified	F n/r	n/r	n/r	At 40 weeks of pregnancy	n/r
Henkin et al. 2016 [21]	2/59 patients with SCAD	C > G change in exon 45 of *COL3A1*	43/F	Nil	n/r	Recurrent dissections of LAD & ramus arteries Note: also diagnosed with FMD at multiple sites	n/r
		C > T change in exon 52 of *COL3A1*	45/F	Yes	Yes	Progressive proximal RCA dissection requiring CABG	Iatrogenic right external iliac dissection during coronary catheterisation
Vandamme et al. 2017 [22]	1/5 patients with SCAD	*COL3A1* variant not specified	28/M	n/r	Yes	NSTEMI – managed conservatively 1 year later - NSTEMI without ischaemic changes on ECG; proximal RCA dissection on angiogram	Initially treated conservatively. 1 year later, PCI and stenting of RCA (led to pseudoaneurysm of radial artery)
Cereda et al. 2017 [36]	1	*COL3A1* variant not specified	43/F	n/r	n/r	Two days post-partum, multivessel SCAD. Coronary angiography: stenosis of the LAD and LCx arteries not reversible with nitrates, OCT: intimal tears of dissection in left main, proximal circumflex and wall haematoma in mid LAD and LCx	1st Stent, 2nd Drug-eluting stent from the ostial LM to the proximal LAD with final kissing-balloon inflation
Kaandan et al. 2018 [23]	3/44 patients with SCAD	c.1859dup p.(Gly621Argfs*8)	30/M	Yes	Yes	SCAD at 30 and second SCAD at 34; no further details	n/r
		c.709 G > A p.(Gly237Arg)	38/M	n/r	Yes	Single SCAD event; no further details	n/r
		c.2212 G > A p.(Gly738Ser)	21/F	n/r	n/r	Single SCAD event	n/r
Shalhub et al. 2019 [16]	Cohort of 86 vEDS patients (2 patients with SCAD)	c.4360 C > T p.(Gln1454*)	45/F	n/r	n/r	n/r	Coronary artery bypass
		c.2337+2 T > C p.Gly762_Lys779del	41/F	n/r	Yes	LAD dissection post colonic rupture	Led to cardiac arrest
Carss et al. 2020 [5]	2/384 patients with SCAD	c.4295 G > T p.(Arg1432Leu)*	41/F	Possible	Yes	Cardiac arrest, multi-segment SCAD, Proximal and Mid LCx dissection	n/r; survived
		c.712 C > T p.(Arg238*)	33/F	Possible	Yes	NSTEMI, multivessel SCAD, LAD and RCA dissection (Distal Involvement, Branch Involvement)	n/r; survived
Carss et al. 2020 [5]	1/92	c.2798dupG p.(Ser934Ilefs*35)	n/r	n/r	None^+^	n/r	n/r
Bos et al. 2021 [37]	1	c.1744 G > A p.(Gly582Ser)	33/F	Nil	None	Chest pain; Angiography showed Type F coronary dissection cause by a Type 4 SCAD of RCA	Percutaneous coronary intervention with placement of four stents
Ekladious et al. 2022 [33]	1	*COL3A1* variant not specified	24/F	Yes	Yes	Chest pain: Angiography showed dissecting aneurysm of RCA	Conservative (Lipitor, metoprolol, ICU observation for 48 hours)
Zekavat et al. 2022 [24]	3/130 patients with SCAD	c.709 G > A p.(Gly237Arg)	n/r	n/r	Yes	n/r	n/r
		c.1330 G > A p.(Gly444Arg)	n/r	n/r	Yes	n/r	n/r
		c.1859dup p.(Gly621Argfs*8)	n/r	n/r	Yes	n/r	n/r
Wang et al. 2022 [25]	1/336 patients with SCAD	c.3898 G > T (p.Glu1300*) (P)	n/r	n/r	n/r	Peripartum SCAD Recurrent SCAD	
Yagi et al. 2022 [28]	1/12	c.2815 G > C, p.(Gly939Arg) (LP)	13/M	n/r	Yes	RCA dissection	n/r
Solyst et al. 2022 [29]	2/88	*COL3A1* variants not specified	n/r	n/r	n/r	n/r	n/r
Erfe et al. 2022 [41]	1	*COL3A1* variant not specified	28/F	n/r	n/r	10 days post-partum Acute chest pain, SOB LAD and obtuse marginal artery dissection	Conservative management; Ischaemic dual papillary muscle rupture resulting in cardiogenic shock requiring prosthetic mitral valve
Li et al. 2022 [40]	1	c.1347+1 G > A	37/M	Nil	Yes	Chest pain: Dissection located between proximal and middle segments of LAD and progression involving LM trunk and LCX	Occlusion of proximal LCx (TIMI flow grade 0), complication of intramural haematoma; died 9 days post-SCAD (pericardial effusion)
Fukuhara et al. 2022 [39]	1	*COL3A1* variant not specified	50/F	n/r	Yes	Multiple episodes of arterial dissection (right renal, splenic, left renal); day 20, presented with chest and back pain and SCAD.	Leaking rupture of RCA and proximal LAD with pericardial effusion. Open-heart surgery, VF and death
Hopfgarten et al. 2022 [38]	1	c.2555 G > T, p.(Gly852Val)	61/F	Nil	Yes	Retrosternal chest pain; dissection of obtuse marginal branch of LCA	Conservative management; Postero-medial papillary muscle rupture with severe MR requiring MVR
DiLiberto et al. 2023 [42]	1	*COL3A1* variant not specified	43/M	Yes	Yes	Chest pain Type 1 SCAD of left main and left anterior descending artery	Stent of LM and LAD; proximal LCx dissection post stenting management with stent replacement. Further dissection and rupture of LAD, plus aortic rupture, cardiac tamponade and death
Yagyu et al. 2013 [31]	1/60 with vEDS	*COL3A1* variant not specified	n/r	n/r	n/r	n/r	n/r
Demirdas et al. 2024 [14]	4: 5/142 (1 prev reported in Bos et al.)	4 of 5 patients had glycine substitutions	3xM/2xFage 39 ^+^/−3.5	Yes	Yes	n/r	n/r
Kumral et al. 2024 [43]	1	c.3221 G > A p. (Gly1074Arg) (P)	36/M	Nil	None	Retrosternal chest pain LAD dissection (TIMI flow 2)	Stents x 2

See Supplementary Table 1.4 for detailed family history and detailed previous history (e.g. easy bruising, joint hypermobility, arterial events).

*21 patients with SCAD but 1 removed as described by Nakamura et al., 2009 [34].

+ (further reported by Tarr et al. 2022 [4]).

*AAA* abdominal aortic aneurysm, *CA* coronary artery, *CABG* coronary artery bypass graft, *CT* computed tomography, *CVD* cardiovascular disease, *CCF* carotid-cavernous fistula, *DVT* deep vein thrombosis, *ECG* electrocardiogram, *FMD* fibromuscular dysplasia, *fh* family history, *F* female, *M* male, *FMD* fibromuscular dysplasia, *FU* follow-up, *HI* haploinsufficiency, *HTN* hypertension, *ICU* intensive care unit, *LAD* left anterior descending, *LCx* left circumflex artery, *LM* left main artery, *LP* likely pathogenic, *MI* myocardial infarction, *MRI* magnetic resonance imaging, *MR* mitral regurgitation, *n/r* not reported, *MVR* mitral valve replacement, *NSTEMI* non-ST–elevation myocardial infarction, *OCT* optical coherence tomography, *P-SCAD* peripartum SCAD, *P* pathogenic, *PCI* percutaneous coronary intervention, *RCA* right coronary artery, *SCAD* spontaneous coronary artery dissection, *SOB* shortness of breath, *STEMI* ST–elevation myocardial infarction, *vEDS* vascular Ehlers-Danlos syndrome, *VF* ventricular fibrillation.

### Retrospective cohort study

#### Study population

In total, 440 patients with vascular EDS were identified through the UK National EDS service. Clinical data was screened for a history of myocardial infarction. Twenty potential SCAD-vEDS cases were selected for more detailed evaluation and this identified six patients for inclusion. Further details of the fourteen patients that were not included in the analysis (S2) are available in Table S2.1 of Supplementary information.

Separately, eight potential SCAD-vEDS cases were identified through a group of 384 individuals in the SCAD Registry that had undergone genomic sequencing as a result of SCAD [5]. Four were identified for inclusion (exclusions available in Table S2.2 of Supplementary information).

A total of ten SCAD-vEDS cases were therefore identified through the UK National EDS service and the UK SCAD registry. Nineteen SCAD-nonvEDS age and sex-matched controls were identified through the SCAD registry (nine SCAD-vEDS cases were matched to two SCAD-nonvEDS controls; in one case, only a single matched control was available due to age). The data from this new case-control group has not been formally analysed in conjunction with the data from the systematic review, because of the significant variability in quality and availability of information in previous publications.

#### Molecular diagnosis

All included cases of vEDS were molecularly confirmed using the ACGS criteria for variant classification [20] and information is provided in Table 2; six patients had class 4 (likely pathogenic) variants, and four patients had class 5 (pathogenic) variants (see Supplementary data Table S2.3 for further analysis of variants). Glycine substitutions were most prevalent (*n* = 8) but variants resulting in haploinsufficiency were also present (*n* = 2) and therefore, due to small sample size, it was not established whether there could be a genotype-phenotype correlation.

**Table 2:** Clinical information regarding spontaneous coronary artery dissection in vEDS-SCAD cohort.

Case	Age of SCAD diagnosis	Other known risk factors	Clinical presentation	Location	Cardiac presentation	Management	Complications
**1**	**29**	Stress, exercise (½ hour before)	Sweaty, clammy, sudden onset chest pain radiating to ears; vomiting	Left circumflex artery and right coronary artery dissection	Inferior ST elevation MI	Stent	Iatrogenic dissection/perforation and contrast extravasation; right iliac artery dissection post-angiogram;
**2**	**35**	29 weeks pregnant	Acute chest pain	Left circumflex artery and left anterior descending artery	NSTEMI	Treated conservatively	Nil
**3**	**50**	Stress (close bereavement 3 days prior)	Sudden onset difficulty in breathing and general feeling unwell	Right coronary artery	n/k	Treated conservatively	Nil
**4**	**40**	5 months post-partum; started ‘boot camp’ 2 days prior	Pain in arms, teeth, jaw; sweating, difficulty in breathing	Left circumflex artery	n/k	Treated conservatively	Nil
**5**	**32**	Performing in a concert; heavy exercise prior (40 push ups)	Sudden onset chest pain whilst singing	Right coronary artery	n/k	Treated conservatively	Nil
**6**	**43**	Smoker (10cpd) Occurred during gym class	Shortness of breath and nausea; chest pain	Right coronary artery	NSTEMI	Stents x 2	Nil
**7**	**33**	Smoker BMI 34 (obese) Recent emotional trigger – bad news Free weights and bootcamp 5 hours prior	Sudden onset chest pain, breathlessness, dizziness, sweating, jaw and arm pain	Left anterior descending artery	NSTEMI	Treated conservatively	Nil
**8**	**46**	Recent emotional stress Treated hypertension BMI 25.6 (overweight)	Sudden onset central chest pain, breathlessness and sweating	Left main stem into left anterior descending artery and diagonal branch	Anterior STEMI	Treated conservatively	Nil
**9**	**45**	Recent emotional stress BMI 31.5 (obese) Sleep-deprivation due to long shift at work	Sudden onset chest pain radiating to neck, breathlessness, flushing, palpitations, nausea, dizziness and sweating	Right coronary artery	NSTEMI	Treated conservatively	Nil
10*	**42**	Physical exertion (putting heavy tent up) Recent emotional stress Sleep-deprivation	Chest tightness, numbness in left arm, nausea, dizziness and sweating	Right coronary artery	NSTEMI followed 5 days later by inferior STEMI	Initially treated conservatively (no angiogram); after 2^nd^ ECG changes, had angiography	Resulted in catheter-induced dissection which spiralled down RCA. Stent distal RCA just proximal to bifurcation and intervening segment

^*^Brother of case 10 presented age 43 with acute chest pain. He was aware of his sister’s diagnosis of SCAD and as he had a confirmed diagnosis of vEDS (previous left iliac artery aneurysm, splenic artery aneurysm (embolization and thrombosis), bilateral iliac dissections and femoral dissection), he declined angiography on admission. He has therefore not been included in this study but had features highly suggestive of SCAD (troponin rise, NSTEMI, normal CT aorta and good response to conservative management). There were no complications, and he made a good recovery.

#### Clinical features

Available clinical characteristics of ten patients in the current SCAD-vEDS cohort are summarised in Table 2 (one patient was not seen in EDS service and therefore full information was not available). The median age at vEDS diagnosis was 41 years (age range 28–51) and median age at last follow-up was 46 years (age range 38–60). Two out of ten patients were male, one of whom was the oldest in the cohort at the time of his SCAD. Six out of nine cases had a family history of an arterial event but only one case (case 10) had been diagnosed with vEDS prior to SCAD due to familial testing. Otherwise, the SCAD event resulted in the initiation of genetic testing in the clinical setting in six out of seven cases. The seventh patient was diagnosed five years after their SCAD event (as a result of another family member being diagnosed with vEDS). Only one case (case 1) had a previous known history of complications relating to aneurysms (de novo *COL3A1* variant) and in this case, the diagnosis of vEDS had also been made prior to the SCAD event [45].

On detailed history taking and examination, seven out of nine patients had other features relating to vEDS to a varying degree. However, case 5 and 9 had no minor features of vEDS, lacking even subtle features such as susceptibility to bruising. There were no deaths in our cohort.

Nine out of ten patients have undergone extra-cardiac imaging (minimum neck to pelvis) after their diagnosis of vEDS. The results were variable but entirely normal in two patients.

Details of the SCAD event are summarised in Table 3. The median age of SCAD was 41 years (age range 29–50). One patient was pregnant; another was 5 months post-partum and exercised strenuously two days prior to the onset of symptoms. Six patients had had a recent emotional event and six were also carrying out intense exercise at the time or just prior to their event.

**Table 3:** Clinical information relating to vEDS for SCAD-vEDS cohort (*n* = 10).

Case	Age at last FU/Sex	Age of vEDS diagnosis	vEDS known at time of SCAD event	Variant (pathogenicity)	Family History of vEDS/vEDS features	vEDS features	Imaging results
1	44 y/F	28	Yes	c.2492 G > A p.(Gly831Asp) ACMG: PM2, PS4_mod, PM1_str, PP3 (class 4)	No	Unilateral CDH; easy bruising; age 9 anterior cruciate ligament rupture; age 27 haemorrhagic rupture of liver cyst; age 28 right peroneal artery aneurysm; age 29 coronary rupture during angiography, right iliac artery dissection post-angiogram; multiple courses of PGD/IVF – age 32 splenic artery aneurysm rupture after hormonal stimulation; age 38 dissection of infra-renal aorta complicated by sigmoid volvulus; age 44, right-sided infra-renal dissection	Age 44: CTA - Iliac artery aneurysms
2	43 y/F	38	No	c.2177 G > T p.(Gly726Val) ACMG: PM2, PS4_mod, PM5_str, PM1_str, PP3 (class 5)	No	Easy bruising, hypermobility, prominent eyes;	Age 42: MRA neck to pelvis - normal
3	60 y/M	51	No	c.382 C > T p.(Gln128*) ACMG: PM2, PVS1 (class 5	Yes	Varicose veins – 20 s; easy bruising;	Age 59: MRA neck to pelvis - infrarenal aortic aneurysm 4.5 cm; common iliac 2.7 cm
4	45 y/F	41	No	c.2329 G > C p.(Gly777Arg) ACMG: PM2, PS4_sup, PM1_str, PP3 (class 4)	Yes	Easy bruising, facial features of vEDS, joint pain, TMJ dislocation;	Age 44: MRA neck to pelvis - normal
5	38 y/M	34	No	c.1996G>A p.(Gly666Ser) ACMG: PM1_str, PM5, PP3 (class 4)	Yes	Nil	Age 37: MRA neck to pelvis - 9 mm pseudoaneurysm right vertebral artery and tortuous splenic artery
6	47 y/F	43	No	c.970 G > A p.(Gly324Ser) ACMG: PM2, PS4_mod, PM1_str, PP3 (class 4)	Yes	Uterine prolapse; hysterectomy with significant prolapse	Age 43: MRA neck to pelvis – normal Age 46: CT – small aneurysm of left vertebral artery; 3 mm increase in aortic root dimension in 3 years and 5 mm
7	40 y/F	-	NA	c.712 C > T p.(Arg238*) ACMG: PM2, PVS1 (class 5)	Unknown	Scoliosis; subconjunctival haemorrhages	Age 33: MRA brain to pelvis – tortuous anatomy of cervical arteries; tonsillar left carotid artery
8	48 y/F	48	No	c.2959 G > A p.(Gly987Ser) ACMG: PM2, PS4_mod, PM1_str, PP3 (class 4)	Yes	Amniotic band syndrome (fingers) with aged appearance to hands, unilateral congenital talipes, easy bruising, distal hypermobility and subluxations, thinning of scalp hair, facial features, skin translucency, flat feet	Age 47: CT thorax and abdomen – mildly dilated aortic root (41 mm at level of PA) CTA head and neck enlarged dysplastic right internal carotid artery with a small pseudoaneurysm at C1 level; also possible narrowing at level of left V4 segment of vertebral artery – suspicious of an old vertebral artery dissection (h/o associated trauma)
9	50 y/F	50	No	c.2627 G > C p.(Gly876Ala) ACMG: PM1_str, PM2_mod, PP3 (class 4)	Yes (at time of review, but not at time of SCAD event)	Nil	Unknown
10	52 y/F	41	Yes	c.1618 G > A p.(Gly540Arg) ACMG:PM1_str PM2, PS4_mod, PP3, PP4 (class 5)	Yes	Achilles’ tendon tears and dislocations Bilateral varicose veins Easy bruising Sparse hair	Age 42: medial splenic artery aneurysm; renal artery aneurysm; mild dilatation in lower internal carotid and carotid bulb

*FU* follow up, *CDH* congenital dislocation of hip, *PGD* preimplantation genetic diagnosis, *IVF* in vitro fertilisation, *MRI* magnetic resonance imaging, *CTA* computerised tomography angiography.

#### Angiographic analysis

All ten SCAD-vEDS individuals were included in the blinded angiogram analysis with 19 SCAD-nonvEDS. For one case, (case 5) only one randomly selected age- and sex-matched control was available. All other cases had two matched controls. Case versus control analysis is described in Table 4.

**Table 4:** Case (SCAD-vEDS) versus control (SCAD-nonvEDS) angiographic analysis.

Characteristic	Cases (*n* = 10)		Controls (*n* = 19)	*P* values, calculated with Fisher’s exact test (two-tailed)
**Location of SCAD**				
Left main coronary artery	1 (7.7%)		0	0.3448
Left anterior descending artery	3 (23.1%)		18 (75%)	0.0005
Left circumflex artery	3 (23.1%)		2 (8.3%)	0.3064
Right coronary artery	6 (46.1%)		4 (16.6%)	0.0514
**AHA coronary segment involved**				
Proximal	5 (29.4%)		5 (14.7%)	0.2439
Mid	5 (29.4%)		10 (29.4%)	1
Distal	6 (35.3%)		15 (44.1%)	0.3904
Branch	1 (5.9%)		4 (11.8%)	0.6328
**More than one vessel involved**	3 (30%)		4 (21.1%)	0.6647
**More than one segment in the vessel involved**	5 (50%)		9 (47.4%)	1
**Saw angiographic classification**				
Type 1	3 (30%)		2 (10.5%)	0.3064
Type 2	5 (50%)		14 (73.7%)	0.2439
Type 3	2 (20%)		0	0.1108
Type 4	0		3 (15.8%)	0.532
**Initial TIMI grade flow**				
0 (no flow)	0	2 (10.5%)		0.532
1	0	1 (5.3%)		1
2	1 (10%)	3 (15.8%)		1
3 (good flow)	9 (90%)	13 (68.4%)		0.3667
**Tortuosity index (median)**	1	1		1
**Type of intervention**				
Conservative	7 (70%)	12 (63%)		1
Stent	3 (30%)	5 (26.3%)		1
Balloon	0	0		n/a
Wiring	0	2 (10.5%)		0.532
**Number of stents for those stented**				
1	1 (33.3%)	2 (40%)		1
2	1 (33.3%)	0		0.3448
3	0	3 (60%)		0.532
5	1 (33.3%)	0		0.3448
**Complications**	2 (20%)	4 (21.1%)		1

Location of SCAD was variable; isolated right coronary artery (RCA) dissection occurred in five cases and isolated LAD and left circumflex artery (LCx) each occurred in one case. The LAD, the most common reported site for SCAD in the general population was statistically lower in SCAD-vEDS cases compared to SCAD-nonvEDS controls (23.1% versus 75%, *p* value = 0.0005) and RCA was seen at a higher proportion (46.1% versus 16.6%, *p* value = 0.05). Three of the ten vEDS patients had multi-vessel dissections. This is not dissimilar to the SCAD-nonvEDS group (30% in SCAD-vEDS cases versus 21.1% in SCAD-nonvEDS controls). In addition, multi-segmental disease was seen in similar frequencies in both cohorts (50% in vEDS cases and 47.4% in SCAD-nonvEDS controls). Type I Saw classification [46] was seen more in cases compared to controls but not to a significant degree (30% versus 10.5%, *p* value = 0.3).

**Fig. 1:**
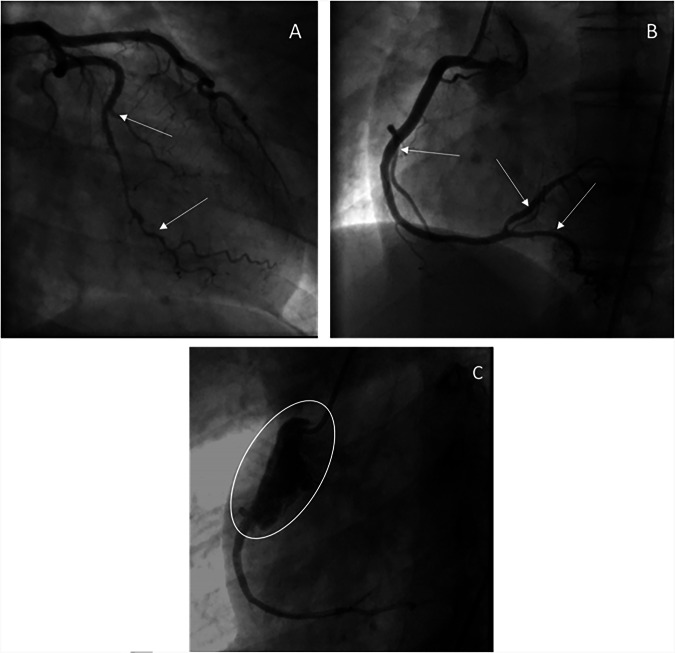
Angiographic findings. • Multivessel dissection of circumflex (**A**—white arrows) and right coronary (**B**—white arrows). • Further angiography was complicated by an extensive iatrogenic dissection/perforation shortly after catheter intubation of the coronary ostium and resulted in a spiral dissection flap contrast extravasation (**C**). • The right coronary artery was treated with a covered stent. The circumflex was managed conservatively. • Post-procedure, she also developed a right iliac artery dissection.

The proportion undergoing intervention was similar in the two groups, as was the complication rate. Three of the SCAD-vEDS cases (cases 1, 6 & 10) underwent stent insertions and two of these cases experienced complications. The two cases where the diagnosis of vEDS was known prior to the SCAD event underwent intervention rather than conservative management. The proportion treated with stenting was very similar to the control group (25% stents in SCAD-vEDS cases versus 26.7% in SCAD-nonvEDS controls). In Case 1, a coronary rupture occurred shortly after catheter intubation of the coronary ostium and extravasation of contrast was evident as was a spiral dissection. After the procedure this patient also developed a right iliac artery dissection (Fig. 1). In Case 10, the RCA was cannulated (femoral artery approach) with initially improved ECG changes but subsequently a catheter-induced dissection in very proximal RCA at catheter tip occurred, which spiralled down RCA leading to a stenosis before bifurcation. The proximal dissection was treated with a stent and distal RCA was also stented just proximal to bifurcation. Three further stents were inserted.

#### Summary of all available cases

Supplement 3.1 summarises the data extracted from the systematic review combined with the current case series.

Using all data available (including cases from the systematic review (where reported) and this retrospective cohort study, male to female ratio was calculated as 1: 1.6 (19 males, 30 females from 49 reported). Age of SCAD event ranged from 13 to 61, a median of 37 and a mean of 36.5 (from 23 reported). In addition, in one report, the ages of SCAD in four individuals were defined as 39 ± 3.5. Whilst presence or absence of family history was recorded in 9 out of 10 in the cohort study, in the systematic review, this was overall poorly reported: in five cases, there was specific mention of no family history suggestive of vEDS; in seven cases, relevant family history was present; in the rest of the cases, there was no mention either way. One reported no family history of sudden death/cardiovascular disease [35]. Specific molecular data was available for 24 individuals. Over half were identified to carry a glycine substitution in the triple helix of *COL3A1* and one-third had variants resulting in haploinsufficiency. Three splice-site variants were reported (13%). The molecular data in Henkin et al. was difficult to interpret [21] and in several cases, whilst the specific *COL3A1* variant was not listed, publications confirmed molecular testing had been completed and vEDS was confirmed with a deleterious *COL3A1* variant.

Seven of the cases were noted to be related to pregnancy or the post-partum period. Other risk factors/ triggering events such as exercise were listed sparsely and therefore not included in the analysis. However, it is noteworthy that three SCAD episodes occurred after surgical intervention for other events related to vEDS e.g. arterial events or hollow organ events. Several cases reported more than one SCAD episode in an individual.

Where angiographic data was included, the RCA and LAD were affected at similar frequency (21/42 compared to 19/42). Multi-vessel SCAD was seen in one-third of events (14/42). Eighteen individuals out of 40, where sufficient data was available, experienced complications related to SCAD, 12 in whom death occurred. One death was in a 13-year-old male [28].

## Discussion

In this study, we sought to identify clinical and angiographic features which might help distinguish patients with SCAD-vEDS from the general SCAD population. We present findings on patients both from a systematic review of the literature and from a UK SCAD-vEDS series and report clinical, angiographic, and molecular features. Establishing an underlying diagnosis of vEDS is crucial to ensure best and appropriate management, especially as it has been shown that treatment and surveillance improves outcome [13] and it may also be relevant for other family members who could be at risk of having vEDS. In addition, awareness of an underlying vEDS diagnosis can be extremely helpful in managing future events including cardiovascular events, especially when considering the potential for complications during invasive interventional or surgical procedures.

We show that reported cases in the medical literature of vEDS and SCAD occurring together are rare. With the new cases presented here, to our knowledge, there are a total of 66 confirmed cases of SCAD-vEDS now in the literature. Limited data is available for some of the historical cases when diagnosis and management of SCAD may have been quite different from current recommendations. It is noteworthy that interventions were more prevalent in the earlier reports, when perhaps there was less awareness of the underlying diagnosis and a preference for a conservative approach to revascularisation where possible for SCAD [1, 2].

Reviewing the available demographic data from the systematic review and our cohort, the median age of reported SCAD was 37.5 (mean of 38). The male to female ratio is 1:1.8. This compares with a median age of 51.7 and a 1:7.7 male to female reported in a prospectively recruited non-genotyped Canadian SCAD population [47]. Although the small sample size precludes meaningful statistical comparison, this provides hints that SCAD may occur at a comparatively younger age and in a higher proportion of males when co-occurring with vEDS. This supports content in the research letter published in 2024 [17].

If reported, environmental triggers e.g., physical and/or emotional stress were seen in SCAD-vEDS, as in the general SCAD populations [2]. Peripartum SCAD (P-SCAD) accounts for around 5% of SCAD cases and is associated with a more severe clinical and angiographic presentation [48]. There are several reports of the SCAD event occurring in vEDS patients during pregnancy (9 out of 66–13.6%). The impact of reporting bias in the cases identified from the systematic review on these factors is unknown.

Including all SCAD-vEDS cases with data on location of SCAD, the RCA appears to be proportionally affected more than in the general SCAD population (*p* = 0.0514), suggesting the anatomical distribution of SCAD may be different in SCAD-vEDS with a lower predilection for the LAD (*p* = 0.005). One-third had multi-vessel dissections, a higher proportion compared to the general SCAD population [49], suggesting a more severe presentation in SCAD-vEDS compared to SCAD-nonvEDS although these findings will require confirmation as larger series become feasible. In the systematic review, while the numbers are more normally distributed, the RCA appears more frequently affected in those with vEDS than is seen in a typical SCAD population. This association may be amplified due to small cohort numbers, however, and even if confirmed, these potential differences may not be sufficient to be clinically useful for individualised prediction of vEDS from the general SCAD population. Between our cohort and the literature review, there are several reports of procedural complications (20 out of 50–40%), although with individual case reports, there may be bias towards reporting complications. In the known cases, complications were all after intervention for the SCAD event, reiterating that a non-invasive approach is more favourable if possible. Three cases of SCAD are known to have occurred after another non-cardiac operative procedure [16, 35, 39]. Both these findings suggest that surgical intervention in vEDS may be a trigger for further events including arterial events such as SCAD. As such, the sibling of case 10 who requested to not have an angiogram because of his known vEDS diagnosis (therefore not included in the study but SCAD confirmed on CTCA) had a good outcome with conservative management. This emphasises the importance of self and family advocacy for rare events such as these and the importance of continuing to disseminate knowledge with regards to the potential for complications associated with vEDS when patients are presenting acutely [50].

Triple helix glycine substitutions were the most common type of variant in our series and the systematic review (62%) as expected in a typical vEDS cohort. An increasing number of rare variants in single genes are beginning to be implicated in cases of SCAD; many of these are genes known to be associated with heritable connective tissue disorders such as *FBN1, LOX* [51] and genes in the TGFβ pathway. Other genes (*TSR1* [52], PTGIR [53], *F11R* [54] and *TLN1* [55, 56]) have also been implicated. In many cases, typical clinical phenotypes are subtle/absent. Twenty percent of our cases had no obvious features of vEDS on detailed history and examination, other than having had a SCAD. Whilst it is recognised that the numbers of patients in our series are small, these studies, along with previous published cases show many patients lack distinguishing features to allow identification of SCAD-vEDS from the wider SCAD population. This is illustrated by the fact that several cases and those in literature were identified on a research basis. This strengthens the argument, despite a relatively low predicted yield of around 10% [57], for comprehensive gene panel testing in cases of SCAD. Currently, no such panel specific for SCAD is recommended in International Consensus statements. Until routine genetic screening of all SCAD patients is made available, those with (i) clinical events pointing to a heritable connective tissue disorder including other arterial aneurysms and/or dissections and hollow organ rupture, or (ii) a family history of SCAD or other clinical events pointing to generalised tissue fragility or (iii) features of a heritable connective tissue disorder on clinical examination as well as features such as younger age and male gender ought to be further evaluated. If a panel for SCAD was to be considered, as well as *COL3A1*, there is potential evidence for several genes to be formally evaluated for inclusion (*FBN1, LOX, TSR1*, *PTGIR*, *F11R* and *TLN1)* [54, 55, 57]. In the advent of advancing genomic technology, access to broader panel testing for the correct indications may result in increased testing and consequently increased molecular diagnoses.

### Limitations

The limitations of systematic reviews are well documented [58]. In this project, the two most significant limitations are: (i) that cases of vascular EDS may not have been identified if the manuscript title and abstract did not include this diagnosis, and (ii) that the quality of the reported data is variable. Not all publications within the systematic review had the specific *COL3A1* variant mentioned and therefore the possibility that some of these may be classified as a variant of uncertain significance (VUS) under current ACGS guidance cannot be excluded.

In terms of the case-control cohort, a significant proportion of patients identified ( >50%) were not eligible for inclusion, mostly due to lack of invasive angiogram availability, as obtaining results of investigations retrospectively is often challenging. One patient was not included as ethics did not allow for recruitment after death. In contrast, in the literature review, there may be bias towards eventful clinical scenarios including death especially with single case reports.

Multi-segmental involvement, coronary tortuosity and Saw classification and TIMI flow grades were not well recorded in previous literature; therefore, this could not be included in the full analysis. We also cannot fully confirm that there are no duplicates in our analysis although variant and other details were compared as much as possible.

Total cohort numbers are small and therefore statistical analysis may not be meaningful, and firm conclusions cannot be drawn from them. Further prospective studies with genotyping are required to confirm the observation of demographic and angiographic differences between SCAD-vEDS and SCAD-nonvEDS. As SCAD is not very common—estimated prevalence 4% of patients presenting with AMI [59] and vEDS is rare (1 in 90,000) [60], a study on the co-existence of the two diagnoses requires long-term collaborative data collection and prospective validation.

## Conclusion

This study demonstrates that although the clinical and angiographic/imaging phenotype of SCAD may be subtly different in vEDS, this is unlikely to be sufficient to distinguish SCAD-vEDS from SCAD-nonvEDS. While detailed history, family history and examination may point towards an underlying vEDS diagnosis, only development of appropriate routine gene panel testing in SCAD patients will ensure identification of known monogenic causes and ensure appropriate management and facilitate predictive genetic testing in relatives. This is particularly important for conditions such as vEDS, where a known diagnosis will strongly influence management decisions leading to improved clinical outcomes in this group of patients.

## Supplementary information

Supplement 1. Systematic Review documents

Supplement 2. New cohort documents

Supplement 3. Combined results from Systematic Review and Cohort

## Data Availability

Data is available upon reasonable request.

## Institutions where work was performed

National Ehlers Danlos Syndrome Service and Leicester British Heart Foundation Centre of Research Excellence.
