# Supplementary material for: Spontaneous coronary artery dissection and vascular Ehlers-Danlos syndrome: a systematic review and case series
Source: Eur J Hum Genet. 2026 Mar 17;34(6):818–30. doi: 10.1038/s41431-026-02074-1 (PMC13246757; doi:10.1038/s41431-026-02074-1)
Supplement: Supplementary file 1 — Supplement 1. Systematic Review documents [file 41431_2026_2074_MOESM1_ESM.docx]

**Supplementary material**

**Appendix 1. Systematic review information**

**S1.1: Search strings used for each database**

**Embase via Ovid**

1            coronary artery dissection/

2            (SCAD or Spontaneous Coronary Artery Dissection* or Spontaneous Coronary Dissection* or Coronary Artery Dissection* or Spontaneous Dissection* or Coronary Dissection* or Arterial dissection* or rupture* or aneurysm*).mp.

3            1 or 2

4            Ehlers-Danlos syndrome, type IV/

5            (vEDS or Vascular EDS or Vascular Ehlers Danlos Syndrome* or EDS IV or IV EDS or Ehlers Danlos Syndrome* IV or IV Ehlers Danlos Syndrome* or sack barabas type ehlers danlos syndrome* or ecchymotic type ehlers danlos syndrome*).mp.

6            4 or 5

7            3 and 6

**Medline via Ovid**

1            (SCAD or Spontaneous Coronary Artery Dissection* or Spontaneous Coronary Dissection* or Coronary Artery Dissection* or Spontaneous Dissection* or Coronary Dissection* or Arterial dissection* or rupture* or aneurysm*).mp.

2            Ehlers-Danlos Syndrome, Type IV/

3            (vEDS or Vascular EDS or Vascular Ehlers Danlos Syndrome* or EDS IV or IV EDS or Ehlers Danlos Syndrome* IV or IV Ehlers Danlos Syndrome* or sack barabas type ehlers danlos syndrome* or ecchymotic type ehlers danlos syndrome*).mp.

4            2 or 3

5            1 and 4

**Maternity and Infant care via Ovid**

1            (SCAD or Spontaneous Coronary Artery Dissection* or Spontaneous Coronary Dissection* or Coronary Artery Dissection* or Spontaneous Dissection* or Coronary Dissection* or Arterial dissection* or rupture* or aneurysm*).mp.

2            (vEDS or Vascular EDS or Vascular Ehlers Danlos Syndrome* or EDS IV or IV EDS or Ehlers Danlos Syndrome* IV or IV Ehlers Danlos Syndrome* or sack barabas type ehlers danlos syndrome* or ecchymotic type ehlers danlos syndrome*).mp.

3            1 and 2

**Scopus**

scad OR "Spontaneous Coronary Artery Dissection*" OR "Spontaneous Coronary Dissection*" OR "Coronary Artery Dissection*" OR "Spontaneous Dissection*" OR "Coronary Dissection*" OR "Arterial dissection*" OR rupture* OR aneurysm*

AND

veds OR "Vascular EDS" OR "Vascular Ehlers Danlos Syndrome*" OR "EDS IV" OR "IV EDS" OR "Ehlers Danlos Syndrome* IV" OR "IV Ehlers Danlos Syndrome*" OR "sack barabas type ehlers danlos syndrome*" OR "ecchymotic type ehlers danlos syndrome*"

**Web of Science**

Topic: scad OR "Spontaneous Coronary Artery Dissection*" OR "Spontaneous Coronary Dissection*" OR "Coronary Artery Dissection*" OR "Spontaneous Dissection*" OR "Coronary Dissection*" OR "Arterial dissection*" OR rupture* OR aneurysm*

AND

Topic: veds OR "Vascular EDS" OR "Vascular Ehlers Danlos Syndrome*" OR "EDS IV" OR "IV EDS" OR "Ehlers Danlos Syndrome* IV" OR "IV Ehlers Danlos Syndrome*" OR "sack barabas type ehlers danlos syndrome*" OR "ecchymotic type ehlers danlos syndrome*"

**S1.2. PRISMA Clinical and angiographic findings in patients with spontaneous coronary artery dissection and vascular Ehlers-Danlos Syndrome**

Studies from databases/registers **(n = 1726)**

Embase (n = 558)

Scopus (n = 415)

Web of Science (n = 389)

MEDLINE (n = 362)

World Health Organization (WHO) (n = 2)

References from other sources **(n = 7)**

Citation searching (n = 7)

**Identification**

References removed **(n = 1050)**

Duplicates identified manually (n = 70)

Duplicates identified by Covidence (n = 980)

Marked as ineligible by automation tools (n = 0)

Other reasons (n = 0)

Studies excluded **(n = 583)**

Studies not retrieved **(n = 0)**

Studies assessed for eligibility **(n = 100)**

Studies sought for retrieval **(n = 100)**

Studies screened **(n = 683)**

Studies excluded **(n = 70)**

Wrong setting (n = 9)

Paper unavailable (n = 1)

Article not in English (n = 6)

vEDS not genetically confirmed (n = 6)

No angiography described/included (n = 1)

SCAD not diagnosed angiographically (n = 2)

No spontaneous coronary artery dissection (n = 45)

**Screening**

Studies included in review **(n = 30)**

**Included**

Included studies ongoing **(n = 0)**

Studies awaiting classification **(n = 0)**


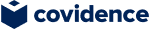


**S1.3: Overview of full text analysis**

SCAD: spontaneous coronary artery dissection

HTAD: Heritable thoracic aortic disease

vEDS: vascular Ehlers Danlos Syndrome

FMD: fibromuscular dysplasia

Papers from full text review n=30

Total = 30

Case series/reports n=14

HTAD cohort studies n=1

SCAD cohort studies n=6

vEDS cohort studies n=9

Total number of cases n=14

Total number of cases n=1

Total number of cases n=16

Total number of cases n=39

Total = 70

Excluded n=1

Included n=13

Excluded n=5

Included n=11

Total included = 56

Total excluded = 14

Included n=1

Excluded n=8

Included n=31

Verstraeten et al., 2020: one case excluded as affected by FMD, not SCAD; second case included in current reported cohort

Wang et al. 2022: three cases excluded as variant of uncertain significance

Angwin et al. 2023: one case excluded as included in current reported cohort

Shalhub et al., 2020: one case expected to be duplication from previous cohort report (Shalhub et al., 2019)

Bowen et al., 2023: one case excluded as patient included in current reported cohort

Demirdas et al., 2024: one case excluded as previously reported in Bos et al., 2021

Pepin et al., 2014: five cases excluded as not confirmed SCAD

**S1.4 Table with detailed Systematic review information**

**Table 4**

| Reference | Number of individuals | Genetics | Age/Sex | Family History | Previous history | Presentation/Risk factors | Management/Complications |
| --- | --- | --- | --- | --- | --- | --- | --- |
| Nakamura et al. 2008 | 1 | c.1988G>A  (p.Gly663Asp) | 33F | Nil | Age 29 – MI, likely due to LAD dissection (diagnosis based on MRI evidence of previous anterior MI)  Age 33 – ruptured splenic artery aneurysm during pregnancy | 4 months post-partum;  Bilateral lower jaw pain; angiography showed dissection of posterolateral branch of LCx  After 2 weeks – further chest pain; angiography showed LAD dissection and occlusion and RCA dissection | Angioplasty to stent LAD.  NB: few hours later, patient died from rupture of right common iliac artery (possibly related to puncture site) |
| Hampole et al. 2011 | 1 | c.31+1G>C | 29/M |  | Diagnosed with vEDS age 24 after ruptured left common iliac artery aneurysm  Age 26 – left renal artery dissection with symptoms. Asymptomatic aneurysms of coeliac, right renal, superior mesenteric, left vertebral, left carotid, hepatic & right common iliac arteries on CT. | Chest pain ;  Angiography showed aneurysm and dissection of LAD | Saphenous vein bypass graft; Post-op VF, resuscitated including chest compressions. Opening of chest identified an aortic rupture (possibly due to chest compressions). Repaired surgically. |
| Leistritz et al. 2011 | 1/82 | *COL3A1* variant not specified | n/r | n/r | n/r | SCAD, no further details | n/r |
| Ohyama et al. 2011 | 1 | p(Gly85Asp)? | 45/F |  | left CCF age 30, headache and tinnitus 14 days before admission discovered to be right CCF | sudden onset of chest pain following transarterial embolization for right carotid-cavernous fistula | Extravasation of contrast material from multiple sites in distal segments of the left circumflex artery and right coronary artery, resulting in pericardial bleed and tamponade. Vessels with ruptured sites were markedly spastic. Second angiography 30 minutes later |
| Pepin et al. 2014 | 20: cohort of 1231 vEDS patients (21 patients with SCAD but 1 removed as described by Nakamura et al.) | n/r | 12x F  8x M | n/r | In some eg. AAA or other arterial aneurysms; esophageal tear during intubation | 9x CA  2x LAD  1x LAD, LM  4x RCA  1x RCA, LAD  1x LCA; RCA  1x LM  1x LC  3 pregnancy-related | 7 died |
| Murray et al. 2014 | 1/526 | *COL3A1* variant not specified | F  n/r | n/r | n/r | At 40 weeks of pregnancy | n/r |
| Henkin et al. 2016 | 2/59 patients with SCAD | C>G change in exon 45 of *COL3A1* | 43F | Nil | n/r | Recurrent dissections of LAD & ramus arteries  Note: also diagnosed with FMD at multiple sites | n/r |
|  |  | C>T change in exon 52 of *COL3A1* | 45F | Family members with vEDS (no details) | Infrarenal aorta and bilateral common iliac artery ectasias | Progressive proximal RCA dissection requiring CABG | iatrogenic right external iliac dissection during coronary catheterisation |
| Vandamme et al. 2017 | 1/5 patients with SCAD | *COL3A1* variant not specified | 28M | n/r | Varicose veins since childhood | NSTEMI – managed conservatively  1 year later - NSTEMI without ischaemic changes on ECG; proximal RCA dissection on angiogram | Initially treated conservatively. 1 year later, PCI and stenting of RCA (led to pseudoaneurysm of radial artery) |
| Cereda et al. 2017 | 1 | *COL3A1* variant not specified | 43/F | n/r | n/r | Two days post-partum; multivessel SCAD: coronary angiography showed a stenosis of the left anterior descending and circumflex arteries that was not reversible with nitrates, OCT showed intimal tears of dissection in left main, proximal circumflex and a wall haematoma in mid LAD and circumflex | 1st Stent, 2nd Drug-eluting stent from the ostial LM to the proximal LAD with final kissing-balloon inflation |
| Kaadan et al. 2018 | 3/44 patients with SCAD | c.1859dup  p.(Gly621Argfs*8) | 30M | Father has diagnosis of vEDS | Translucent skin, easy bruising, varicose veins | SCAD at 30 and second SCAD at 34; no further details | n/r |
|  |  | c.709G>A  p.(Gly237Arg) | 38M | n/r | Non-traumatic retroperitoneal haematoma at age of 35 | Single SCAD event; no further details | n/r |
|  |  | c.2212G>A  p.(Gly738Ser) | 21F | n/r | No details given | Single SCAD event | n/r |
| Shalhub et al. 2019 | Cohort of 86 vEDS patients (2 patients with SCAD) | c.4360C>T  p.(Gln1454*) | 45F | n/r | n/r | n/r | Coronary artery bypass |
|  |  | c.2337+2T>C  p.Gly762_Lys779del | 41F | n/r | Colonic perforation  Arterial event age 36 | LAD dissection post colonic rupture | Led to cardiac arrest |
| Carss et al. 2020 | 2/384 patients with SCAD | c.4295G>T p.(Arg1432Leu)* | 41F | Sibling with recurrent pneumothorax  Another sibling with patellar dislocation | High palate  Pes planus | Cardiac arrest, multi-segment SCAD,  Proximal and Mid LCx dissection | n/r; survived |
|  |  | c.712C>T  p.(Arg238*) | 33F | Mother with scoliosis | Scoliosis, sub-conjunctival haemorrhages | NSTEMI, multivessel SCAD,  LAD and RCA dissection (Distal Involvement, Branch Involvement) | n/r; survived |
| Carss et al. 2020 | 1/92 | c.2798dupG  p.(Ser934Ilefs*35) | n/r | n/r | No clinical characteristics of EDS upon FU (further reported by Tarrs et al. 2022) | n/r | n/r |
| Bos et al. 2021 | 1 | c.1744G>A  p.(Gly582Ser) | 33/F | Nil | No previous history | Chest pain; Angiography showed Type F coronary dissection cause by a Type 4 SCAD of RCA | Percutaneous coronary intervention with placement of four stents |
| Ekladious et al. 2022 | 1 | *COL3A1* variant not specified | 24/F | Sister – bowel rupture (18y);  Brother – two strokes (18y, 19y), dislocation of shoulder and poor wound healing; mother died age 35 during delivery | Two episodes of chest pain age 22 associated with raised troponin and diagnosed as NSTEMI; recurrent meniscal tear of both knees; carotid cavernous fistula (15y); occipital lobe stroke; easy bruising, poor wound healing. | Chest pain:  Angiography showed dissecting aneurysm of RCA | Conservative (Lipitor, metoprolol, ICU observation for 48 hours) |
| Zekavat et al. 2022 | 3/130 patients with SCAD | c.709G>A  p.(Gly237Arg) | n/r | n/r | ‘The range of clinical findings in vascular EDS’ | n/r | n/r |
|  |  | c.1330G>A  p.(Gly444Arg) | n/r | n/r | As above | n/r | n/r |
|  |  | c.1859dup  p.(Gly621Argfs*8) | n/r | n/r | As above | n/r | n/r |
| Wang et al. 2022 | 1/336 patients with SCAD | c.3898G>T (p.Glu1300*) (P) | n/r | n/r | n/r | Peripartum SCAD  Recurrent SCAD |  |
| Yagi et al. 2022 | 1/12 | c.2815G>C, p.(Gly939Arg)  (LP) | 13/M | n/r | haemopneumothorax | RCA dissection | n/r |
| Solyst et al. 2022 | 2/88 | *COL3A1* variants not specified | n/r | n/r | n/r | n/r | n/r |
| Erfe et al. 2022 | 1 | *COL3A1* variant not specified | 28/F | n/r | n/r | 10 days post-partum  Acute chest pain, SOB  LAD and obtuse marginal artery dissection | Conservative management;  Ischaemic dual papillary muscle rupture resulting in cardiogenic shock requiring prosthetic mitral valve |
| Li et al. 2022 | 1 | c.1347+1G>A | 37/M | Nil | Trauma-related splenic and intestinal rupture age 17; trauma-related liver rupture age 33;  Bruising;  Prone to skin abrasions | Chest pain  Dissection located between proximal and middle segments of LAD and progression involving LM trunk and LCX | LCX occlusion of proximal segment with TIMI flow grade 0, complicated by massive intramural haematoma; death 9 days post-SCAD (large pericardial effusion found) |
| Fukuhara et al. 2022 | 1 | *COL3A1* variant not specified | 50/F | n/r | Multiple episodes of arterial dissection (right renal, splenic, left renal) | Multiple episodes of arterial dissection (right renal, splenic, left renal); day 20, presented with chest and back pain and SCAD. | Leaking rupture of RCA and proximal LAD with pericardial effusion. Open-heart surgery, VF and death |
| Hopfgarten et al. 2022 | 1 | c.2555G>T, p.(Gly852Val) | 61/F | Nil | Joint pain and easy bruising | Retrosternal chest pain; dissection of obtuse marginal branch of LCA | Conservative management;  Postero-medial papillary muscle rupture with severe MR requiring mitral valve replacement |
| DiLiberto et al. 2023 | 1 | *COL3A1* variant not specified | 43/M | Sudden death in father; vEDS diagnosed in brother after splentic artery rupture | Hypertension; known vEDS diagnosis | Chest pain  Type 1 SCAD of left main and left anterior descending artery | Stent of LM and LAD; proximal left circumflex dissection post stenting management with replacement of stent. Further dissection of LAD and subsequent rupture, as well as aortic rupture, cardiac tamponade and death |
| Yagyu et al. 2023 | 1/60 with vEDS | *COL3A1* variant not specified | n/r | n/r | n/r | n/r | n/r |
| Demirdas et al. 2024 | 4: 5/142 but 1 prev reported in Bos et al. | 4 of 5 patients had glycine substitutions | 3M/2Fage 39+/-3.5 | 2/5 were family members | SCAD was first event in 3 individuals; 2 had prior vascular event (no details) | n/r | n/r |
| Kumral et al. 2024 | 1 | c.3221G>A p. (Gly1074Arg) (P) | 36/M | No clear fh of vEDS or SCAD | No previous events | Retrosternal chest pain  LAD dissection (TIMI flow 2) | Stents x 2 |

AAA, abdominal aortic aneurysm; CA, coronary artery; CABG, coronary artery bypass graft; CT, computed tomography; CVD, cardiovascular disease; CCF, carotid-cavernous fistula; DVT, Deep Vein Thrombosis; ECG – Electrocardiogram; FMD , fibromuscular dysplasia; fh, family history; F, female; M, male; FMD, Fibromuscular Dysplasia; FU, follow-up; HI, haploinsufficiency; HTN, Hypertension; ICU, Intensive Care Unit; LAD, Left anterior descending; LCx, Left circumflex artery; LM, left main artery; LP, likely pathogenic; MI, myocardial infarction; MRI, magnetic resonance imaging; MR, mitral regurgitation; n/r, not reported; NSTEMI, non-ST–elevation myocardial infarction; OCT, optical coherence tomography; P-SCAD, peripartum SCAD; P, pathogenic; PCI, Percutaneous coronary intervention; RCA, Right coronary artery; SCAD, spontaneous coronary artery dissection; SOB, shortness of breath; STEMI, ST–elevation myocardial infarction ; vEDS, vascular Ehlers-Danlos syndrome; VF, ventricular fibrillation.

*21 patients with SCAD but 1 removed as described by Nakamura et al., 2009^22^
