# Supplementary material for: Spontaneous coronary artery dissection and vascular Ehlers-Danlos syndrome: a systematic review and case series
Source: Eur J Hum Genet. 2026 Mar 17;34(6):818–30. doi: 10.1038/s41431-026-02074-1 (PMC13246757; doi:10.1038/s41431-026-02074-1)
Supplement: Supplementary file 2 — Supplement 2. New cohort documents [file 41431_2026_2074_MOESM2_ESM.docx]

**Supplementary material**

**Appendix 2. New cohort data**

**S2.1 Table of individuals excluded from case control analysis**

| Sex | Reason not included |
| --- | --- |
|  | Angiogram from 2013 had a coronary stent in situ. Original angiogram or report to confirm diagnosis of SCAD not obtainable |
|  | Unable to source angiogram or report |
| F | Invasive angiogram was not performed therefore not possible to confirm SCAD |
| F | SCAD in 2003 and angiogram no longer retained by hospital therefore not able to confirm diagnosis of SCAD |
| F | SCAD no confirmed on angiogram review |
| F | Coronary artery spasm |
| M | diagnosis of an aneurysmal circumflex artery and proximal severe stenosis and very ectatic and aneurysmal right coronary artery but no confirmed SCAD |
| F | Variant of uncertain significance in *COL3A1* therefore molecular diagnosis of vEDS not confirmed |
| M | Patient known to have vEDS at point of SCAD admission (brother of case 10) and asked for non-invasive investigations due to iatrogenic risk. Therefore, SCAD confirmed only on coronary computed tomography, not angiography. |
|  | Variant of uncertain significance in *COL3A1* therefore molecular diagnosis of vEDS not confirmed |
| M | Variant of uncertain significance in *COL3A1* therefore molecular diagnosis of vEDS not confirmed |
| F | Variant of uncertain significance in *COL3A1* therefore molecular diagnosis of vEDS not confirmed |
|  | Did not consent to obtain angiogram |
| F | Ethics did not allow for review of angiogram of deceased patient |

Eight patients were excluded as the diagnosis of SCAD was not proven either after review of angiogram or because angiogram was unobtainable. One patient could not be included in the angiography review as they were deceased, and ethics did not allow for this. Four patients were excluded as the diagnosis of vascular EDS was not molecularly proven (variants of uncertain significance rather than deleterious variants). One patient did not consent to obtaining angiography.

**S2.3 Summary of current ACGS criteria for classification of genetic variants (adapted from Durkie et al., 2024**^1^**, Ellard et al., 2020**^2^ **and Richards et al., 2015**^3^**)**

**Table of variants from each of the 10 cases**

| **Case Number** | **Variant in *COL3A1*** | **ACGS criteria used** |
| --- | --- | --- |
| **1** | c.2492G>A p.(Gly831Asp) | PM2, PS4_mod, PM1_str, PP3 (class 4) |
| **2** | c.2177G>T p.(Gly726Val) | PM2, PS4_mod, PM5_str, PM1_str, PP3 (class 5) |
| **3** | c.382C>T p.(Gln128*) | PM2, PVS1 (class 5) |
| **4** | c.2329G>C p.(Gly777Arg) | PM2, PS4_sup, PM1_str, PP3 (class 4) |
| **5** | c.1996G>A p.(Gly666Ser) | PM1_str, PM5, PP3 (class 4) |
| **6** | c.970G>A p.(Gly324Ser) | PM2, PS4_mod, PM1_str, PP3 (class 4) |
| **7** | c.712C>T p.(Arg238*) | PM2, PVS1 (class 5) |
| **8** | c.2959G>A p.(Gly987Ser) | PM2, PS4_mod, PM1_str, PP3 (class 4) |
| **9** | c.2627G>C p.(Gly876Ala) | PM1_str, PM2_mod, PP3 (class 4) |
| **10** | c.1618G>A p.(Gly540Arg) | PM1_str PM2, PS4_mod, PP3, PP4 (class 5) |

|  | **Benign** | | **Pathogenic** | | | |
| --- | --- | --- | --- | --- | --- | --- |
| **Data type** | **Strong** | **Supporting** | **Supporting** | **Moderate** | **Strong** | **Very strong** |
| **Population data** | BA1/BS1  Allele frequency is greater than expected for disorder/ above 5%  BS2  Observation in healthy adult controls in disorders with full penetrance at an early age |  |  | PM2  Absent in controls or very low frequency if recessive in GAD | PS4  Statistically increased prevalence in cases over controls (matched where possible) |  |
| **Computational and predictive data** |  | BP4  Multiple lines of evidence suggest no impact on gene/ gene product  BP1  Missense variant in gene where primarily truncating variants are known to cause disease  BP7  Silent variant with no predicted splicing impact and nucleotide is not highly conserved; splice variants where RNA studies confirm no impact  BP3 In-frame deletion/insertions in a repetitive region without known function | PP3  Multiple lines of computational evidence support a deleterious effect on the gene/ gene product | PM4  Protein length changes as a result of in-frame deletions/insertions in a non-repeat region or stop-loss variants  PM5  Novel missense variant at amino acid residue where a different pathogenic missense change has been seen before | PS1  Same amino acid change as an established pathogenic variant, and splicing variants with the same motif with identical predicted effect | PVS1  Predicted null variant (nonsense, frameshift, specific splice sites, exon deletions, initiation codon disruption) where loss of function is known mechanism of disease; and non-canonical splice variants where RNA analysis confirms aberrant transcription |
| **Functional data** | BS3  Well established functional studies (in vivo or in vitro) show no deleterious effect on protein function |  | PP2  Missense variant in gene with low rate of benign missense variantion and in which missense variants are a common mechanism of disease | PM1  Located in a mutational hot-spot or well-established functional domain without benign variation | PS3  Well established functional studies (in vivo or in vitro) supportive of a damaging effect on gene/gene product |  |
| **Segregation data** | BS4  Non-segregation with disease |  | PP1  Co-segregation with disease in multiple affected family members in known disease-causing gene |  |  |  |
| ***De novo* data** |  |  |  | PM6  Assumed *De novo* (without confirmed maternity/paternity) | PS2  *De novo* (confirmed maternity/paternity) |  |
| **Allelic data** |  | BP2  Observed *in trans* with a dominant variant  Observed *in cis* with a pathogenic variant |  | PM3  For recessive disorders, detected in trans with a pathogenic variant |  |  |
| **Other database** |  | BP6  Reputable source without shared data | PP5  Pathogenic reported in reputable source but evidence is not available to laboratory to perform an independent evaluation |  |  |  |
| **Other data** |  | BP5  Found in case with alternate cause | PP4  Patient phenotype or family history is highly specific for a disease with a single genetic aetiology |  |  |  |

BS, benign strong; BP, benign supporting; GAD, Genome Aggregation Database; PM, pathogenic moderate; PP, pathogenic supporting; PS, pathogenic strong; PVS, pathogenic very strong

Variants are classified as pathogenic (class 5), likely pathogenic (class 4), variant of uncertain significance (class 3), likely benign (class 2) and benign (class 1) according to specific combinations of these data points. For example, pathogenic variants can be determined from a combination of one pathogenic very strong and either one or more strong, one or more moderate or two or more supporting (Ellard et al., 2020, Durkie et al., 2024) ^1,2^.

1 Durkie M, Cassidy E-J, Berry I, *et al.* ACGS Best Practice Guidelines for Variant Classification in Rare Disease 2024. , 2024.

2 Ellard S, Baple EL, Callaway A, *et al.* ACGS Best Practice Guidelines for Variant Classification in Rare Disease 2020. *Association for Clinical Genomic Science* 2020.

3 Richards S, Aziz N, Bale S, *et al.* Standards and guidelines for the interpretation of sequence variants: A joint consensus recommendation of the American College of Medical Genetics and Genomics and the Association for Molecular Pathology. *Genetics in Medicine* 2015. doi:10.1038/gim.2015.30.

**S2.2 Patient enrolment and exclusion in reported cohort**

Assessed for eligibility

(n = 28)

…)

Excluded (n = 18)

No angiographic data (n = 8)

SCAD not confirmed on angiogram

(n = 4)

Variant of uncertain significance (VUS) in *COL3A1* therefore vEDS not confirmed*

(n = 4)

No consent (n = 1)

Unable to consent under ethics as patient deceased (n = 1)

*VUS reported:

1. c.4306G>C; p.(Ala1436Pro)

2. c.4295G>T; p.(Arg1432Leu)

3. c.2333A>G; p.(Asp778Gly)

4. c.42404G>C; p.(Ala1402Pro)

Enrolled in study

(n = 10)
