# Supplementary material for: Spontaneous coronary artery dissection and vascular Ehlers-Danlos syndrome: a systematic review and case series
Source: Eur J Hum Genet. 2026 Mar 17;34(6):818–30. doi: 10.1038/s41431-026-02074-1 (PMC13246757; doi:10.1038/s41431-026-02074-1)
Supplement: Supplementary file 3 — Supplement 3. Combined results from Systematic Review and Cohort [file 41431_2026_2074_MOESM3_ESM.docx]

Supplementary table 3.1 Summary of data from systematic review combined with current case series.

|  | **Systematic review** | **Current reported cohort** | **Total** |
| --- | --- | --- | --- |
| **Total number of cases** | 56* | 10 | 66 |
| Demographic data (where available) | | | |
| **Males** | 19 | 2 | 21 |
| **Females** | 30 | 8 | 38 |
| **Age range for SCAD** | 13-61 (mean 36.5, median 37) | 29-50 (mean 39.5, median 41) | 13-61 (mean 38 years; median 37.5) |
| **Relevant family history** | 7 (6 no suggestive family history) | 6 | 13 out of 23 |
| **Pregnancy related** | 7 | 2 | 9 |
| Molecular data (where available) | | | |
| **Total available variants** | 24 | 10 |  |
| Glycine substitution | 13 | 8 | 21/34 |
| Haploinsufficiency | 8 | 2 | 10/34 |
| Splice site variant | 3 | 0 | 3/34 |
| Angiographic data (where available, in some cases more than one set of data in an individual) | | | |
| **Single vessel** |  |  |  |
| LAD only | 7 | 1 | 8 |
| RCA only | 9 | 5 | 14 |
| LCx only | 4 | 1 | 5 |
| Left main artery only | 1 | 0 | 1 |
| **Multi-vessel** |  |  |  |
| RCA and LAD | 4 | 0 | 4 |
| RCA and LCx | 2 | 1 | 3 |
| LAD and LCx | 1 | 1 | 2 |
| LAD and LM | 2 | 1 | 3 |
| LAD, LCx, LM | 2 | 0 | 2 |
| Outcome data (where available) | | | |
| **Complications** | 18 | 2 | 20 |
| Further coronary dissection | 0 | 2 | 2 |
| Further coronary dissection and aortic rupture | 1 (resulted in death) | 0 | 1 |
| Other arterial complications | 4 (iliac artery rupture x 2, aortic rupture x 1, pseudoaneurysm of radial artery) (1 resulted in death) | 0 | 4 |
| Death – unknown cause | 8 | 0 | 8 |
| Other complications | 5 (pericardial bleed, 2 x papillary muscle rupture, intramural haematoma, pericardial effusion)  (2 resulted in death) | 0 | 5 |

*Although a total of 56 cases, data is only reported where available
